# Clinical decision-making to facilitate appropriate patient management in chiropractic practice: *'the 3-questions model'*

**DOI:** 10.1186/2045-709X-20-6

**Published:** 2012-03-14

**Authors:** Lyndon G Amorin-Woods, Gregory F Parkin-Smith

**Affiliations:** 1 Murdoch University, School of Chiropractic and Sports Science, South Street, Murdoch, 6150 Perth, Western Australia; 2 Murdoch University Chiropractic Clinic, Murdoch University, South Street, Murdoch, WA 6150 Perth, Australia

**Keywords:** Chiropractor, Clinical decision-making, Differential diagnosis, Red flags, 3-questions model

## Abstract

**Background:**

A definitive diagnosis in chiropractic clinical practice is frequently elusive, yet decisions around management are still necessary. Often, a clinical impression is made after the exclusion of serious illness or injury, and care provided within the context of diagnostic uncertainty. Rather than focussing on labelling the condition, the clinician may choose to develop a defendable management plan since the response to treatment often clarifies the diagnosis.

**Discussion:**

This paper explores the concept and elements of defensive problem-solving practice, with a view to developing a model of agile, pragmatic decision-making amenable to real-world application. A theoretical framework that reflects the elements of this approach will be offered in order to validate the potential of a so called '3-Questions Model';

**Summary:**

Clinical decision-making is considered to be a key characteristic of any modern healthcare practitioner. It is, thus, prudent for chiropractors to re-visit the concept of defensible practice with a view to facilitate capable clinical decision-making and competent patient examination skills. In turn, the perception of competence and trustworthiness of chiropractors within the wider healthcare community helps integration of chiropractic services into broader healthcare settings.

## Background

"The exclusivity of medical knowledge and skill is being broken down. Inter-professional learning is now commonplace in medical education and seems likely to increase. Professional boundaries are being blurred as more and more things that were once the sole domain of doctors are being undertaken by other health care professionals. None of us works alone any longer, but in multidisciplinary teams in which we depend upon the expertise of others. This is not a diminution of medicine, but a strengthening of health care. We must acknowledge that, more than ever before, knowledge is available to patients and the public."

Sir Graeme Catto, Past-President of the UK General Medical Council (2005) [1]

Skills and competence in clinical decision-making are taught in various undergraduate healthcare programmes and cultivated through clinical experience. Many working diagnoses or clinical impressions, especially those for musculoskeletal (MSK) presentations, are developed through a process of exclusion, particularly when the context of diagnosis is uncertain. In other words, a definitive diagnosis may be elusive; however, a decision on management is still required.

Therefore, clinical decision-making centres on fundamental principles:

1. Avoiding patient harm (i.e., "Is it safe?"), and

2. Providing effective care (i.e., "Will it work?" and, "Are the necessary resources available?").

It should be noted at the outset that the challenge of reaching a meaningful diagnosis is by no means unique to chiropractors. Neuro-musculoskeletal presentations are notoriously difficult to diagnose precisely where labels (for MSK) are often arbitrary [2-4]. Terms such as 'wear and tear', 'degeneration', 'stress', 'arthritis', 'slipped disc', 'lumbago', 'cricked neck' and similarly ambiguous and vague terms are anecdotally still commonly encountered in practice.

Regarding low back pain, for example, the uncertainty was highlighted by the taxonomy subcommittee of the International Association for the Study of Pain (IASP) led by Bogduk (2000); "The only intellectually and clinically honest diagnosis for most cases of low back pain (is) lumbar spinal pain of unknown or uncertain origin [5]." (As an aside, it is debatable, in the opinion of the authors, whether this "intellectually and clinically honest" diagnosis adds superior clarity to that of the traditional chiropractic specific term "subluxation").

Initial decisions by chiropractors on management made on the basis of exclusion of 'red flags' constitute a defensive approach, particularly for non-specific spinal pain. As primary contact practitioners, chiropractors will invariably see patients with serious disorders yet to be diagnosed, such as emerging myocardial infarction, stroke, venous thrombo-embolism and aortic aneurysm, among others. In approximately 80% of these cases, the signs and symptoms of deterioration evolve over a few hours (sometimes days) or are episodic in nature, before obvious symptoms or complications occur [6]. Yet, these clinical presentations are often either ignored, missed or a diagnosis delayed, potentially leading to a life-threatening situation due to late treatment [7]. 'Red flags' may be implicated in as many as 1-5% of cases of spinal pain [8].

Competent clinical decision-making in chiropractic practice, therefore, not only revolves around common presentations, such as spinal pain or headache, but also covers the screening, examination, and potential triage of presentations that may necessitate referral to other healthcare providers. Differential diagnosis is part of this process of contemporary clinical decision-making expected of primary contact healthcare disciplines, of which formulating a diagnosis or clinical impression is a feature. Some chiropractors may view this approach taught in chiropractic undergraduate programmes as the 'medicalisation' of chiropractic, however, differential diagnosis is actually less about treating disease and more about the adoption of rational, clinical decision-making. By implication, the defensive approach to differential diagnosis alluded to earlier, where a definitive label is unlikely, offers a model for effective decision-making that is sensible, practical, and user-friendly and also congruent with traditional chiropractic practice.

### Development of the *3-questions model*

This paper explores the concept and elements of defensive problem-solving practice, with a view to developing a model of agile, pragmatic decision-making amenable to real-world application. A theoretical framework that reflects the elements of this approach and evaluation against obligatory standards of practice (Australia and the United Kingdom) will be offered in order to validate the potential of the *3-Questions Model*.

The chiropractic profession, particularly in Western countries, finds itself in a rapidly evolving healthcare landscape, with 'modernisation' being a consequence of escalating costs, an aging population, and an ever-diminishing relative resource base [9]. With a view to rationalising resources health system decision-makers are increasingly vigilant about the delivery of safe, evidence-based, cost-effective care, summarised as "the right care at the right time in the right place" [10,11]. With this imperative in mind, the authors propose three straightforward questions that frame clinical decision-making within the context of diagnostic uncertainty.

#### Question 1: What is the likelihood I will delay access to more appropriate care for this patient?

Contemporary chiropractic offers an array of conservative treatments, selected and delivered on the basis of research evidence, tradition, expertise, patient preference, or a combination of all these. Yet, before any treatment can be applied, the practitioner has a duty of care to ensure that the patient will receive the most appropriate care at the right time, which may in fact necessitate referral to another healthcare provider. In other words, the preferences, traditions, and care philosophy of the practitioner are secondary to the needs of the patient, supported by the maxim; 'first do no harm' [12,13]. In short, the practitioner must consider what has gone wrong with the patient as a whole to bring them to this point, and what role the chiropractor may play in their management [12].

The favourable natural history and episodic nature of uncomplicated spine-related disorders, the well documented 'Hawthorne Effect', combined with a low risk of adverse effects, may lull the chiropractor into a false belief that their favoured technique provides a panacea [14]. Indeed, the belief that simply finding, quantifying and addressing 'subluxations' is the only role of the chiropractor ignores the reality that patients with serious or life threatening conditions can, and do, present to chiropractors. Also, over-confidence in their care philosophy and treatment technique may lead to the inappropriate treatment of specific patient populations. The failure to recognise these and consequently refer constitutes a significant proportion of malpractice litigation against chiropractors [15].

Chiropractors, along with all other health professionals, must be vigilant concerning the so called 'red flags' for serious illness, and be familiar with the signs and symptoms of emerging ill health or injury (13). Therefore, the practitioner must make several decisions on behalf of each patient on each visit. Firstly; whether the patient can be managed without collaboration with any other health practitioner, secondly; if the patient should be managed in conjunction with other healthcare provider(s), or thirdly; whether the patient should be rejected outright for chiropractic care.

It is relevant to note that various regulatory bodies *expect *inter-professional relationships to exist in the best interest of the patient, for example:

The Australian Code:

Section 5

*5.1 Good care is enhanced when there is mutual respect and clear communication between all health professionals involved in the care of the patient *[16].

The UK code:

Section D1

b) *Identifying where it might be appropriate to consider co-managing the patient with another healthcare practitioner*

c) *Referring patients to other healthcare practitioners when their needs are beyond your own knowledge, skills and competence *[17].

#### Question 2: Is my proposed treatment safe? (What is the likelihood of making this patient worse?)

The risk of adverse events with chiropractic care is enviably low. Even those sceptical of chiropractors and spinal manipulation admit that adverse side effects after chiropractic spinal manipulation are usually mild and transient [18,19]. Combined with high levels of patient satisfaction, the low risk of manual and manipulative therapy reinforces the role of chiropractic in the management of spine-related disorders [20,21]. Consequently, spinal manipulation is now recommended for consideration in recent guidelines for management of uncomplicated spinal pain [8].

Chiropractic practice embraces a broad clinical spectrum including manual, manipulative, soft-tissue, physiological and rehabilitative therapies - choosing which of these to apply, and when, have evolved over time as part of the so called 'Art of Chiropractic'. Based on the likely anatomical source of the presenting complaint, the chiropractor must advise the most appropriate clinical intervention, in the best interest of the patient [12].

Again, it is pertinent to note the requirements of regulators, whereby chiropractors must follow a patient-centred approach that minimises the risk of harm. Question 2 ensures compliance with the following sections:

The Australian code:

Section 2.2 Good care

d) *Practising patient-centred care, including encouraging patients to take interest in, and responsibility for the management of their health and supporting them in this*

f) *Considering the balance of benefit and harm in all clinical management decisions *[16].

The UK Code:

S2.6 Clinical decision making

When drawing up the working diagnosis or rationale for care, you must consider:

a) *Relevant information about the natural history and prognosis of any complaint the patient has*

b) *The potential benefits and risks of care, including contraindications*

c) *The likelihood of recurrence or need for long-term management *[17].

#### Question 3: Do I have all the information I need to answer the first two questions?

Cleland describes the concept of a 'treatment threshold', whereby the clinician gathers information from the health history and the physical examination and further tests until they decide whether or not to provide care for the patient [22]. For the chiropractor, this is not as simple as checking they are breathing and have a spine! The health history and subsequently the relative value of findings from physical examination, orthopaedic tests, and available technologies are assessed according to sensitivity and specificity. Synthesis of the information gathered through history-taking, examination and evaluation of the patient is necessary to formulate a clinical impression, even if the diagnostic context is uncertain. Matters of safety, appropriateness, and cost-effectiveness of treatment are all paramount in the decision-making process and gathering information via thorough patient assessment permits the answering of the third question [23,24].

The Australian Code:

Section 4.2 Good practice involves

a) *A full and thorough assessment of patients using tools, tests and procedures that are appropriate for the gathering of information necessary to form a reasonable diagnosis *[16].

The UK Code:

S2.6 Clinical decision making You must:

a) *Evaluate the patient's health and health needs*

b) *Arrive at and document a working diagnosis or rationale for care, based on the evaluation of the information*

Groopman (2007) advises patients to ask their doctor three pertinent questions which astute chiropractic practitioners would do well to ask themselves on behalf of their patient; "What else could it be?", "Is there anything that doesn't fit?" and "Is it possible there is more than one problem?" The patient may well in the chiropractors' opinion, have a biomechanical (functional) spinal lesion, but what *else *might they have [25,26]?

Thus, the imperative of the clinical encounter is to ensure the patient is in the right place at the right time; more appropriate care is not likely to be delayed, the intervention is not likely to result in harm, and the clinician has enough information to decide. If the cause of the presentation can be labelled (diagnosed), are there co-morbidities, and if so are they significant? The context of the clinical decision is *"what are the ramifications of being incorrect?" *Notwithstanding the traditional chiropractic emphasis on specificity, the reality is the implications for the patient are greater if the clinician 'misses' serious illness or injury compared to making an incorrect MSK diagnosis.

## Discussion

### The 3-questions model of clinical decision making for chiropractors

1. *What is the likelihood I will delay access to more appropriate care for this patient?*

2. *Is my proposed treatment safe? (What is the likelihood of making this patient worse?)*

3. *Do I have enough information to answer the first two questions?*

The chiropractic profession is now highly regulated in developed countries, with standards that demand competent, safe practice and a duty of care to each and every patient. Logically, if chiropractors demonstrate these attributes, their reputation and trustworthiness is enhanced within the healthcare community, facilitating the future integration of chiropractic services into multidisciplinary healthcare settings. The Australian Productivity Commission's *Australia's Health Workforce Report *(2005) contains far-reaching recommendations related to national regulation and registration, workforce rationalisation, and an evolution of the role of all health professionals, with these recommendations driving healthcare transformation [27]. In fact, this transformation is arguably a revolution and presents as an opportunity for chiropractors to potentially take on a more generic role within this 'task substitution', in line with the healthcare workforce requirements of the future [28,29]. In the opinion of the authors this requires a clear demarcation in the role of the chiropractor: being cognisant of differentiation between 'primary contact' and 'primary care'.

The competence of chiropractors to arrive at a meaningful diagnosis remains a somewhat controversial topic. It is well-documented that chiropractors outperform other health professionals in musculoskeletal competence, however, their role in making accurate, cross-professionally relevant diagnoses, in areas other than musculoskeletal health is less clear [30,31]. The ruling in a recent court case in Texas illustrates an interesting paradox [32]. The opinion of the judge that chiropractors could only make a diagnosis in the context of musculoskeletal/biomechanical conditions at first reading is seemingly in contrast to the UK and Australian chiropractic regulatory agencies, where a chiropractor may be disciplined for *failing *to make a diagnosis [16,17]. However, this may not be as inconsistent with the traditional role of chiropractors as it first seems. The chiropractor is not expected, and should not make a diagnosis outside their scope of practice; this is generally the role of the medical practitioner.

The need is emphasised for chiropractors to adopt a consistent decision-making process where the information gathered from patient assessment is synthesised in order to make reasonable judgements about appropriateness and safety of care, particularly when a diagnosis is equivocal. In these circumstances the clinical imperative may not be about having the *right diagnosis*, but rather about generating a *defensible *patient management plan and making prompt referral when necessary- this is the context in which the *3-Questions Model *may be helpful to practitioners.

In short, the authors' contend that while chiropractors should always aim to make as accurate a diagnosis as possible within their scope of practice, recognise this is often not achievable. The focus rather is on forming a defendable clinical rationale for management. This pre-supposes exclusion of those patients outside the clinical remit. This defensive methodology is given further traction by Haldeman (2011) who suggests that "by discarding the term diagnosis, we may be able to spare patients extensive and expensive testing that leads to findings that do not correlate with each other and that do not allow for any generally agreed-on treatment approach"[33]. Society and regulators expect chiropractors to practice safely and competently within their scope of chiropractic practice - this scope of care has traditionally consisted of spinal care that excluded pharmacological or surgical prescription [34-36]. The observations of Catto (2005) offered as the introduction to this paper whilst uttered in reference to medical practitioners are none-the-less certainly relevant to chiropractors.

### Summary

Clinical decision-making is without doubt a key characteristic of any modern healthcare practitioner. It is prudent for chiropractors to re-visit the concept of competent, defensible practice with a view to facilitate clinical decision-making and patient examination skills within the profession. In turn, this improves the reputation and perceived trustworthiness of chiropractors among healthcare providers and facilitates the integration of chiropractic services into broader healthcare settings.

Adept clinical decision-making, even when a definitive diagnosis is elusive, should be the hallmark of chiropractic practice, consequently ensuring that patients are managed safely and appropriately. Thus, the *3-Questions Model *as described in this paper is proposed as a reference-point for pragmatic decision-making by chiropractors.

## Abbreviations

CBA: Chiropractic board of Australia; EG: Evidence based guidelines; GCC: General chiropractic council; IASP: International association for the study of pain; MSK: Musculoskeletal; PICO: Patient intervention comparison outcome; UK: United Kingdom.

## Competing interests

The authors declare that they have no competing interests.

## Authors' contributions

LAW contributed the initial concept and equally to the composition of the paper. GPS contributed equally to the composition of the paper. All authors read and approved the final manuscript.

**Figure 1:**
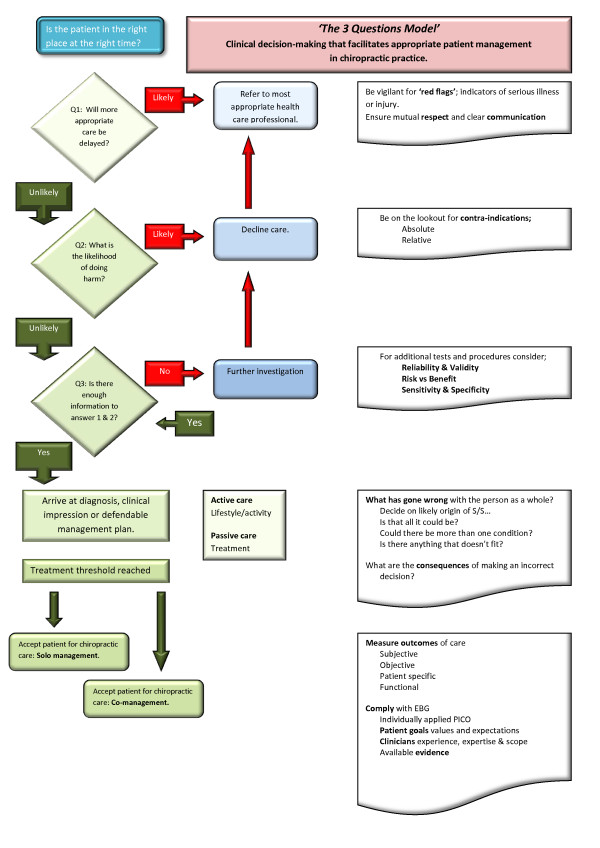
**Algorithm of the 3 Questions Model**.
